# Study on the Effect of Alloying Element Cr on the Corrosion Behavior of Co-Fe-Ni-Mo-W High-Entropy Alloys in Seawater Environments

**DOI:** 10.3390/ma19214585

**Published:** 2026-10-29

**Authors:** Jiaer Lu, Mengqi Yao, Jiang Yu, Yuwan Tian

**Affiliations:** 1 School of Mechanical Engineering, Guangdong Ocean University, Zhanjiang 524088, China; 2 Guangdong Provincial Ocean Equipment and Manufacturing Engineering Technology Research Center, School of Mechanical Engineering, Guangdong Ocean University, Zhanjiang 524088, China

**Keywords:** high-entropy alloys, Cr, seawater, passive film, corrosion products

## Abstract

In marine environments, critical components are susceptible to corrosion failure due to the influence of chloride ions. High-entropy alloys have garnered widespread attention owing to their superior comprehensive properties. This study investigates the corrosion behavior of Co-Fe-Ni-Mo-W high-entropy alloys in seawater and explores the role of chromium (Cr) in modulating their corrosion resistance. Electrochemical methods were employed to compare the corrosion resistance of CoFeNiMo_0.1_W_0.2_ and CoFeNiMo_0.1_W_0.2_Cr_0.7_ high-entropy alloys in seawater, while modern physical characterization techniques—including XRD, SEM, and XPS—were utilized to elucidate the underlying corrosion resistance mechanisms. The results demonstrate that during prolonged immersion in seawater, the surface of the Cr-free CoFeNiMo_0.1_W_0.2_ alloy fails to develop a stable protective layer, leading to severe surface corrosion, with corrosion products including crystalline Ni(OH)_2_ among others. In contrast, the Cr-containing CoFeNiMo_0.1_W_0.2_Cr_0.7_ alloy exhibits excellent self-passivation characteristics in seawater: its open-circuit potential remains within a relatively positive range throughout a 49 d immersion period, its charge transfer resistance increases by nearly two orders of magnitude compared to the Cr-free counterpart, and no significant pitting pits or corrosion product accumulation are observed on its surface. The corrosion resistance mechanism can be attributed to the introduction of Cr, which promotes the formation of a dense passive film enriched with Cr_2_O_3_ on the alloy surface. This passive film possesses superior physical barrier and charge-shielding properties, thereby significantly inhibiting the adsorption and penetration of aggressive Cl^−^ ions, and thus effectively protecting the substrate.

## 1. Introduction

With the rapid development of marine resource exploitation and ocean engineering, metallic structural components are subjected to increasingly harsh service environments characterized by high concentrations and strong permeability of corrosive chloride (Cl^−^) ions, sufficient dissolved oxygen, and alternating wet-dry conditions. These aggressive factors frequently induce severe localized corrosion, such as pitting and crevice corrosion, ultimately leading to premature structural failure [1,2]. Consequently, developing advanced marine engineering alloys with exceptional long-term corrosion resistance remains a critical scientific and technological challenge.

High-entropy alloys (HEAs), as a revolutionary paradigm in alloy design, diverge from traditional single-principal-element systems by exploiting high-entropy effects, severe lattice distortion, and sluggish diffusion. These unique core effects impart HEAs with extraordinary tunability in mechanical properties and microstructural stability [3,4,5]. Among various candidates, transition metal-based HEAs (such as the Co–Fe–Ni matrix systems) have attracted widespread attention due to their stable solid-solution structures and favorable mechanical strength. Previous studies have demonstrated that minor additions of Mo can effectively strengthen the metallic matrix and enhance resistance to localized breakdown, while the incorporation of W contributes to high-temperature solid-solution strengthening [6,7]. However, our preliminary investigations and recent findings indicate that the base CoFeNiMo_0.1_W_0.2_ system lacks highly efficient self-passivating constituents. Upon prolonged exposure to marine environments containing aggressive Cl^−^ ions, its surface oxide film is highly vulnerable to penetration and localized breakdown, resulting in sustained active dissolution and an inability to provide long-term thermodynamic protection.

To overcome this intrinsic limitation, micro-alloying or compositional optimization is imperative to induce spontaneous passivation. Chromium (Cr), as the cornerstone element of conventional stainless steels [8,9], possesses a remarkably low passive film formation energy barrier and a high thermodynamic propensity for spontaneous passivation, making it the most effective additive for constructing dense, protective passive films. Although numerous studies have explored Cr-containing HEAs, the specific capability, dynamic evolution, and microscopic regulation mechanisms by which a controlled amount of Cr modulates the long-term corrosion resistance and passive film stability of the CoFeNiMo_0.1_W_0.2_ matrix in seawater environments remain systematically unrevealed.

Driven by this scientific motivation, this study investigates the comparative corrosion behavior of the base CoFeNiMo_0.1_W_0.2_ and Cr-doped CoFeNiMo_0.1_W_0.2_Cr_0.7_ high-entropy alloys during a long-term (49-day) natural seawater immersion test. Periodic electrochemical impedance spectroscopy (EIS) and open-circuit potential (OCP) measurements were systematically conducted to elucidate the corrosion kinetics and the degradation-resistance mechanisms. Furthermore, advanced characterization techniques—including X-ray photoelectron spectroscopy (XPS), X-ray diffraction (XRD), scanning electron microscopy (SEM), and energy-dispersive X-ray spectroscopy (EDS)—were integrated to decode the chemical state, composition, and physical barrier properties of the surface passive films. This work aims to establish a clear correlation between Cr-induced microstructural passivation and long-term marine corrosion resistance, thereby providing theoretical guidance for the rational design of advanced marine-resistant high-entropy alloys.

## 2. Experimental Methods

### 2.1. Materials

The materials used in the experiment were the high-entropy alloys CoFeNiMo_0.1_W_0.2_ and CoFeNiMo_0.1_W_0.2_Cr_0.7_, prepared from metal particles of Co, Fe, Ni, Mo, Cr, and W with purity exceeding 99.95 wt.%, mixed in stoichiometric ratios. The prepared charge was placed in a vacuum arc furnace, repeatedly inverted and melted five times to ensure compositional homogeneity; after melting, the charge was cooled in the furnace.

The ingot was machined via wire cutting into a specimen measuring 10 mm × 10 mm × 3 mm. The back side of the specimen was welded with a copper wire and then encapsulated with epoxy resin, leaving only the 10 mm × 10 mm working surface exposed. The working surface was sequentially grit-washed using SiC abrasive paper (400#–2000#), followed by ultrasonic cleaning in anhydrous ethanol and deionized water for 10 min each, then air-dried with a cold air blower before being placed in a desiccator for subsequent use.

All chemical reagents used in the experiment (including anhydrous ethanol, inorganic salt reagents—NaCl, MgCl_2_, Na_2_SO_4_, CaCl_2_, etc.—for preparing ASTM D1141-98 [10] simulated seawater, as well as the pH-adjusting reagent NaOH) were procured from Shanghai Aladdin Biochemical Technology Co., Ltd. (Shanghai Chenhua, Shanghai, China), all of which were of analytical grade (AR) and were not further purified prior to use.

### 2.2. Corrosion Resistance Testing Experiment

Artificial seawater prepared in accordance with the ASTM D1141-98 standard (pH adjusted to approximately 8.2) was used as the immersion corrosion medium, and the experimental temperature was set at room temperature. The working surfaces of the encapsulated specimens were fully immersed in the test solution for a total immersion duration of 49 days.

Periodic non-destructive electrochemical tests were performed on the experimental samples using a standard three-electrode cell, employing a high-entropy alloy with a bare area of 1 cm^2^ as the working electrode, a saturated calomel electrode (SCE) as the reference electrode, and a platinum electrode as the auxiliary electrode. Open-circuit potential (OCP) and electrochemical impedance spectroscopy (EIS) measurements were conducted using a CHI 660 electrochemical workstation (Shanghai Chenhua, Shanghai, China) at experimental intervals of 1, 2, 3, 7,14, 21, 28, and 49 days. Prior to the EIS measurements, the OCP was monitored until its fluctuation amplitude fell below 5 mV per 10 min; subsequently, EIS measurements were performed using the steady-state open-circuit potential as the baseline, with an excitation signal amplitude of 10 mV and a frequency range of 100 kHz–10 mHz. The acquired impedance data were then fitted using ZView v3.60 software to extract interfacial kinetic parameters [11].

### 2.3. Passive Film Detection Experiment

To elucidate the chemical composition and elemental valence state distribution of the surface passive film of the two high-entropy alloys after 1 days of full immersion in seawater, X-ray photoelectron spectroscopy (XPS, ESCALAB 250Xi, Scientific, Waltham, MA, USA) was employed to analyze the surface composition of the specimens. The measurements were conducted using a monochromatized Al K_α_ radiation source (*hv* = 1486.6 eV) at a working voltage of 15 kV, with a spot size of 500 μm. The acquired data were charge-corrected using the contaminant C 1s peak (binding energy reference: 284.8 eV), and peak deconvolution analysis was performed using XPSPEAK or Avantage v6 software after subtracting the Shirley background.

### 2.4. Surface Corrosion Morphology Testing Experiment

After immersing the two specimens in simulated seawater for 1 day, the surface microstructural damage morphology of the specimens following immersion and corrosion was examined using scanning electron microscopy (SEM, Carl Zeiss AG, Oberkochen, Germany, equipped with an Oxford Instruments EDS system, Abingdon, UK), and the elemental composition and elemental distribution in characteristic regions were analyzed using an energy-dispersive X-ray spectrometer (EDS). The phase structure of the substrate and surface corrosion products of the specimens before and after immersion was investigated using an X-ray diffraction instrument (XRD), employing a Cu-K_α_ radiation source, with a 2θ range of 5–90° and a scanning rate of 2°/min.

## 3. Experimental Results

### 3.1. Corrosion Behavior

**Figure 1:**
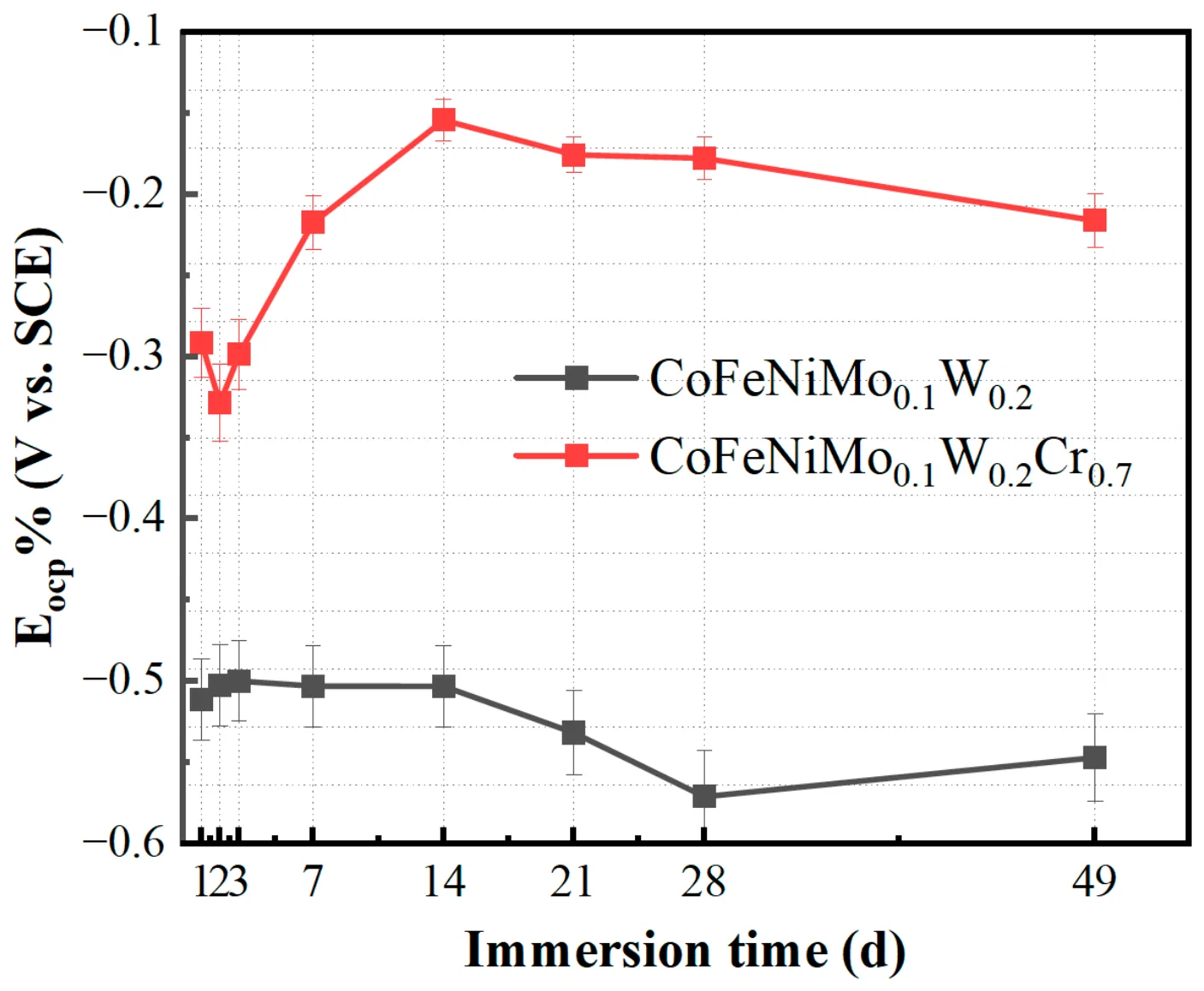
Evolution curves of open-circuit potential (E_ocp_) of CoFeNiMo_0.1_W_0.2_ and CoFeNiMo_0.1_W_0.2_Cr_0.7_ high-entropy alloys during immersion in seawater for 49 days.

Figure 1 shows the evolution of open-circuit potential (OCP) of the CoFeNiMo_0.1_W_0.2_ and CoFeNiMo_0.1_W_0.2_Cr_0.7_ high-entropy alloys during immersion in seawater for 49 days. OCP can be used to characterize the thermodynamic tendency of metal corrosion; a negative shift in OCP indicates an increased thermodynamic tendency for corrosion, whereas a positive shift signifies enhanced corrosion resistance [12]. As observed in Figure 1, during the initial immersion stage (1–7 days), the OCP of the CoFeNiMo_0.1_W_0.2_ high-entropy alloy remained at approximately −0.5 V vs. SCE, reflecting a high thermodynamic susceptibility to active dissolution. As the immersion time extended beyond 7 days, the OCP decreased further and gradually, suggesting that the surface film formed on the alloy struggled to provide effective thermodynamic stability, leading to an increased tendency for matrix dissolution. Overall, the Cr-free CoFeNiMo_0.1_W_0.2_ alloy exhibited a higher thermodynamic tendency toward active dissolution and struggled to maintain a long-term stable protective passive film in seawater. In contrast, the OCP of the CoFeNiMo_0.1_W_0.2_Cr_0.7_ alloy was −0.3 V vs. SCE at the beginning of immersion and progressively shifted positively to −0.15 V vs. SCE between days 1 and 14, demonstrating a gradual increase in corrosion resistance. Throughout the 49-day immersion period, the OCP of the Cr-containing high-entropy alloy remained significantly higher than that of the Cr-free counterpart (by approximately 300–400 mV). These results conclusively demonstrate that the introduction of the alloying element Cr significantly promotes the formation of a surface protective passive film, endowing the substrate with superior thermodynamic corrosion resistance.

**Figure 2:**
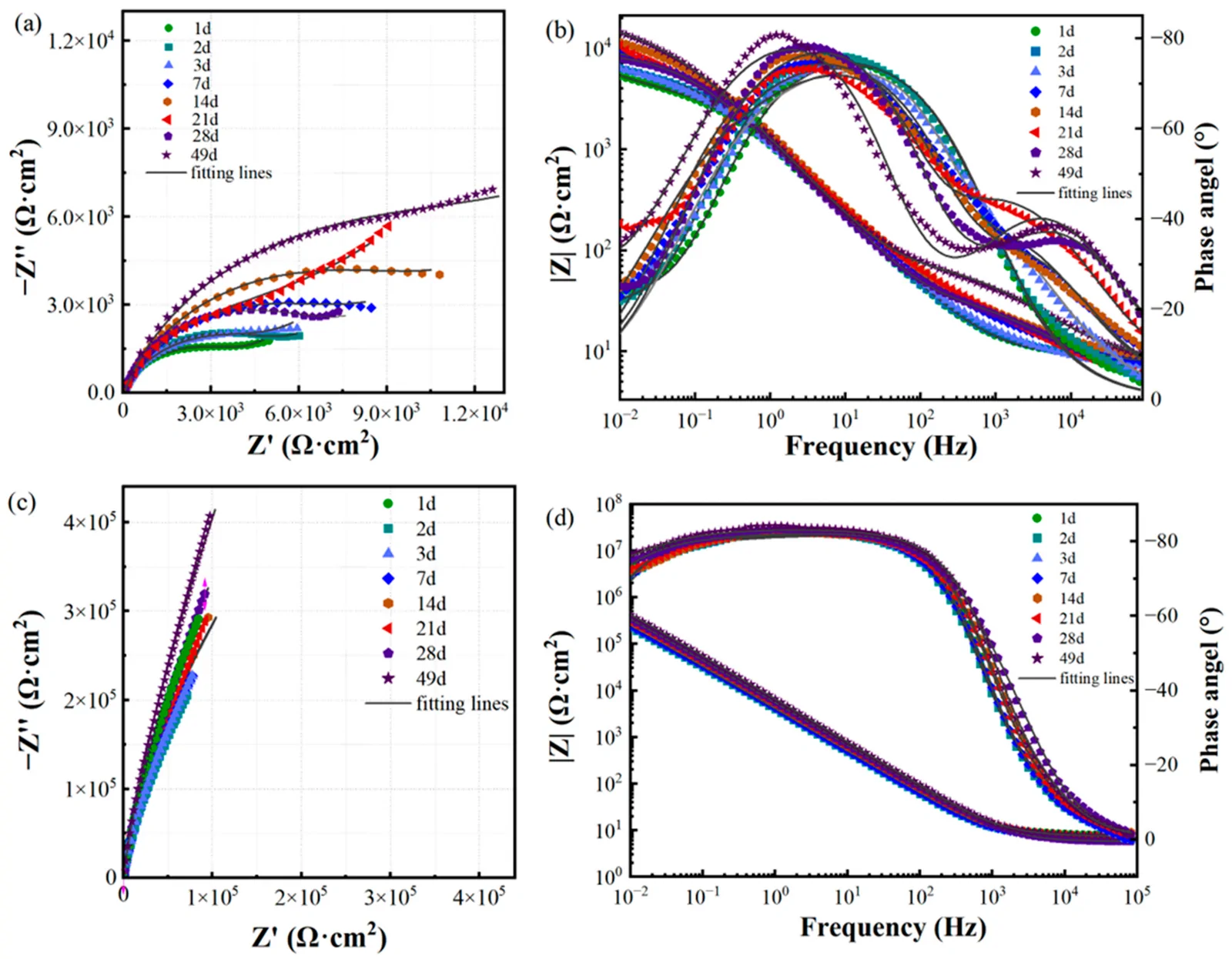
Evolution of EIS spectra for the high-entropy alloys CoFeNiMo_0.1_W_0.2_ and CoFeNiMo_0.1_W_0.2_Cr_0.7_ during 49 days of immersion in seawater: (**a**) Nyquist plot of CoFeNiMo_0.1_W_0.2_; (**b**) Bode plot of CoFeNiMo_0.1_W_0.2_; (**c**) Nyquist plot of CoFeNiMo_0.1_W_0.2_Cr_0.7_; (**d**) Bode plot of CoFeNiMo_0.1_W_0.2_Cr_0.7_.

To systematically evaluate the stability of interfacial passive films and the corrosion evolution behavior of the two high-entropy alloys during 49 days of seawater immersion, electrochemical impedance spectroscopy (EIS) measurements were periodically conducted, with the resulting spectra presented in Figure 2.

For the Cr-free CoFeNiMo_0.1_W_0.2_ alloy (Figure 2a,b), the reactance arc radius in its Nyquist plot remained consistently at a low level throughout the immersion period; in the Bode plot, the low-frequency impedance modulus |Z| at 0.01 Hz fluctuated only around 10^4^ Ω·cm^2^, indicating minimal resistance to active dissolution of the metallic substrate. The phase angle curve in the Bode plot exhibited a dual-peak characteristic across a wide frequency range, suggesting the presence of a dual-time-constant response at the interface: the mid-frequency region corresponds to the response of the outer porous corrosion product (or passive) layer, while the low frequency region corresponds to charge transfer processes at the alloy/electrolyte interface. The maximum phase angle of the alloy ranged only −80°, significantly deviating from ideal capacitive behavior.

Upon introduction of Cr, the impedance spectroscopy characteristics of the CoFeNiMo_0.1_W_0.2_Cr_0.7_ alloy underwent significant changes (Figure 2c,d). Its Nyquist plot exhibits a large-radius capacitive arc, while in the Bode plot, the low-frequency impedance modulus exceeds 10^5^ Ω·cm^2^, representing an increase of nearly two orders of magnitude compared to the CoFeNiMo_0.1_W_0.2_ alloy. The phase angle in the Bode plot displays a broad peak, resulting from the superposition of two peaks, with the maximum phase angle stabilizing between −80° and −85°, approaching that of an ideal capacitor. Combined with the absence of corrosion signs on the sample surface, these findings collectively indicate that the passive film of the CoFeNiMo_0.1_W_0.2_Cr_0.7_ alloy possesses excellent protective properties.

**Figure 3:**
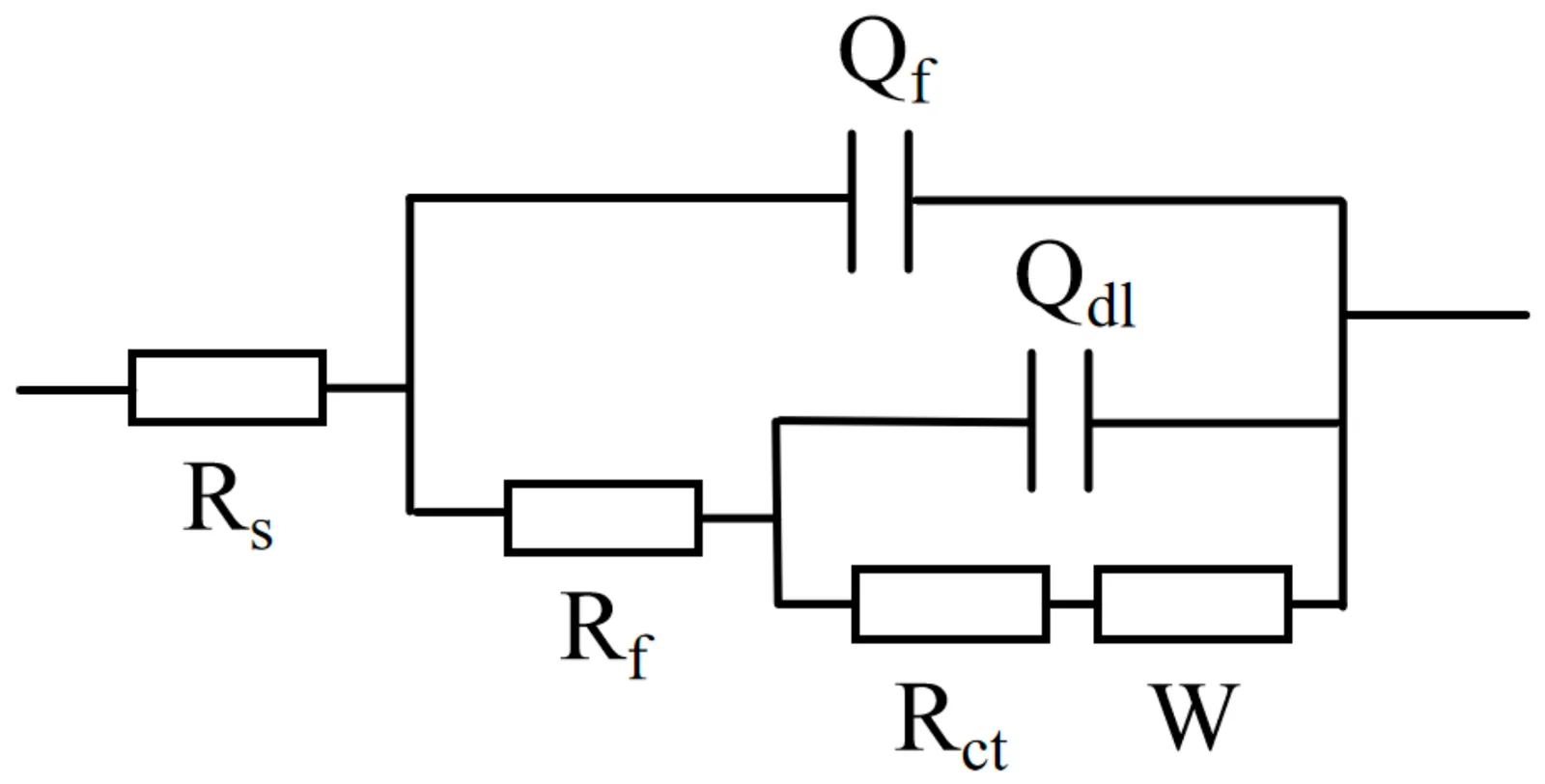
Equivalent circuit model R_s_(Q_f_(R_f_(Q_dl_(R_ct_W)))) for fitting the EIS spectra of high-entropy alloys in seawater medium.

To quantitatively extract the electrochemical process kinetic parameters of high-entropy alloys during full seawater immersion cycles, a two-time-constant nested equivalent circuit model—R_s_(Q_f_(R_f_(Q_dl_(R_ct_W)))) (as shown in Figure 3)—was employed for fitting, based on the mid-frequency double-layer charge transfer and low-frequency diffusion mass transfer characteristics observed in the impedance spectra [13,14,15]. Here, R_s_ denotes the solution resistance; Q_f_ and R_f_ represent the constant-phase-element (CPE) and resistance of the corrosion product layer or passive film, respectively; Q_dl_ and R_ct_ represent the double-layer capacitance and charge transfer resistance at the interface between the alloy substrate and the solution; and W denotes the Warburg impedance characterizing diffusion. In practical corrosion electrode systems, the geometric micro-roughness and pore structure on the electrode surface often lead to deviations from ideal capacitive behavior; therefore, a constant-phase-element (CPE) is used in place of an ideal capacitor, with the following impedance expression [14,15]:

(1) $$Z_{\mathrm{CPE}}=\frac{1}{Y_{0}{\left(J\omega \right)}^{n}}$$

where

*ω*—angular frequency (rad/s);
*J*—imaginary unit (*J*^2^ = −1);
*Y*_0_—admittance magnitude of the constant phase element (Ω^−1^⋅s*^n^*⋅cm^−2^);
*n*—dispersion exponent (0 < *n* ≤ 1), which characterizes the non-ideality of the interfacial capacitance at the electrode interface. As the value of n approaches 1, the interface becomes increasingly dense and smooth, exhibiting behavior that approximates an ideal capacitor; deviations of n from 1 reflect diffusion effects induced by the micro-roughness of the electrode surface, porous defects, and uneven distribution of reaction products; when n equals 0.5, the result corresponds to Warburg impedance. The electrochemical kinetic parameters of the two high-entropy alloys after varying immersion durations, obtained via fitting with the aforementioned equivalent circuit, are presented in Table 1.

As shown in Table 1, the charge transfer resistance (R_ct_) of both alloys is several orders of magnitude larger than the film resistance (R_f_), indicating that charge transfer is significantly hindered during the corrosion kinetics process. In contrast, the surface film layers of CoFeNiMo_0.1_W_0.2_ and CoFeNiMo_0.1_W_0.2_Cr_0.7_ are relatively loose, porous, or thin, resulting in lower resistances. During the initial immersion period (1–7 days), the R_ct_ of the CoFeNiMo_0.1_W_0.2_ alloy remains at a low level of 10^3^–10^4^ Ω·cm^2^, suggesting rapid active dissolution of the substrate. After 49 days of immersion, although R_ct_ increases slightly to 1.48 × 10^4^ Ω·cm^2^, this increase is primarily attributed to the accumulation and thickening of a rough, porous corrosion product layer on the surface. Overall, the CoFeNiMo_0.1_W_0.2_ base alloy fails to form a dense and stable protective passive film in seawater, making it difficult to resist the sustained penetration and degradation by aggressive Cl^−^ ions.

In contrast, the R_ct_ of the Cr-containing CoFeNiMo_0.1_W_0.2_Cr_0.7_ alloy exhibits a remarkable order-of-magnitude improvement, with its charge transfer resistance R_ct_ stabilizing at the 10^6^ Ω·cm^2^ range (e.g., ranging from 1.17 × 10^6^ to 5.25 × 10^6^ Ω·cm^2^, as shown in Table 1), which is two to three orders of magnitude higher than that of the base alloy without Cr addition [16]. This phenomenon is closely attributed to the fact that Cr promotes the formation of a highly dense and stable passive film on the alloy surface, thereby significantly elevating the activation energy barrier for anodic dissolution of the metallic substrate.

Furthermore, from the perspective of interfacial capacitance characteristics, the diffusion indices n1 and n2 of the CoFeNiMo_0.1_W_0.2_Cr_0.7_ alloy both stabilize at values close to 1 (primarily ranging from 0.86 to 0.99, Table 1), which are significantly larger than those of the parent alloy; meanwhile, the imaginary parts of the constant-phase-angle admittance parameters, Y_01_ and Y_02_, are markedly lower than those of the parent alloy. These findings indicate that the introduction of the alloying element Cr results in a homogeneous, dense passive film with minimal defects, effectively inhibits localized dissolution or pitting initiation on the substrate, and significantly reduces interfacial micro-roughness and non-uniformity. This further demonstrates that the corrosion damage is minimal, and that the passive film exhibits excellent long-term protective performance.

In summary, the addition of the alloying element Cr to CoFeNiMo_0.1_W_0.2_ significantly inhibits the initiation and propagation of pitting corrosion of the substrate induced by chloride ions, thereby enhancing its long-term seawater corrosion resistance.

To further evaluate the corrosion kinetics, the equivalent corrosion current density (*i*_corr_) was estimated from the charge transfer resistance (*R*_ct_) obtained from the EIS fitting results, using the Stern-Geary equation (*i_corr_* = *B*/*R_ct_*, with the constant *B* taken as 26 mV). The calculated *i*_corr_ values for the CoFeNiMo_0.1_W_0.2_Cr_0.7_ alloy significantly decreased by two to three orders of magnitude compared to the Cr-free counterpart, further demonstrating that the addition of Cr markedly suppresses the active dissolution kinetics and enhances the corrosion resistance (as listed in Table 2).

### 3.2. Surface Characterization Results After Prolonged Immersion

**Figure 4:**
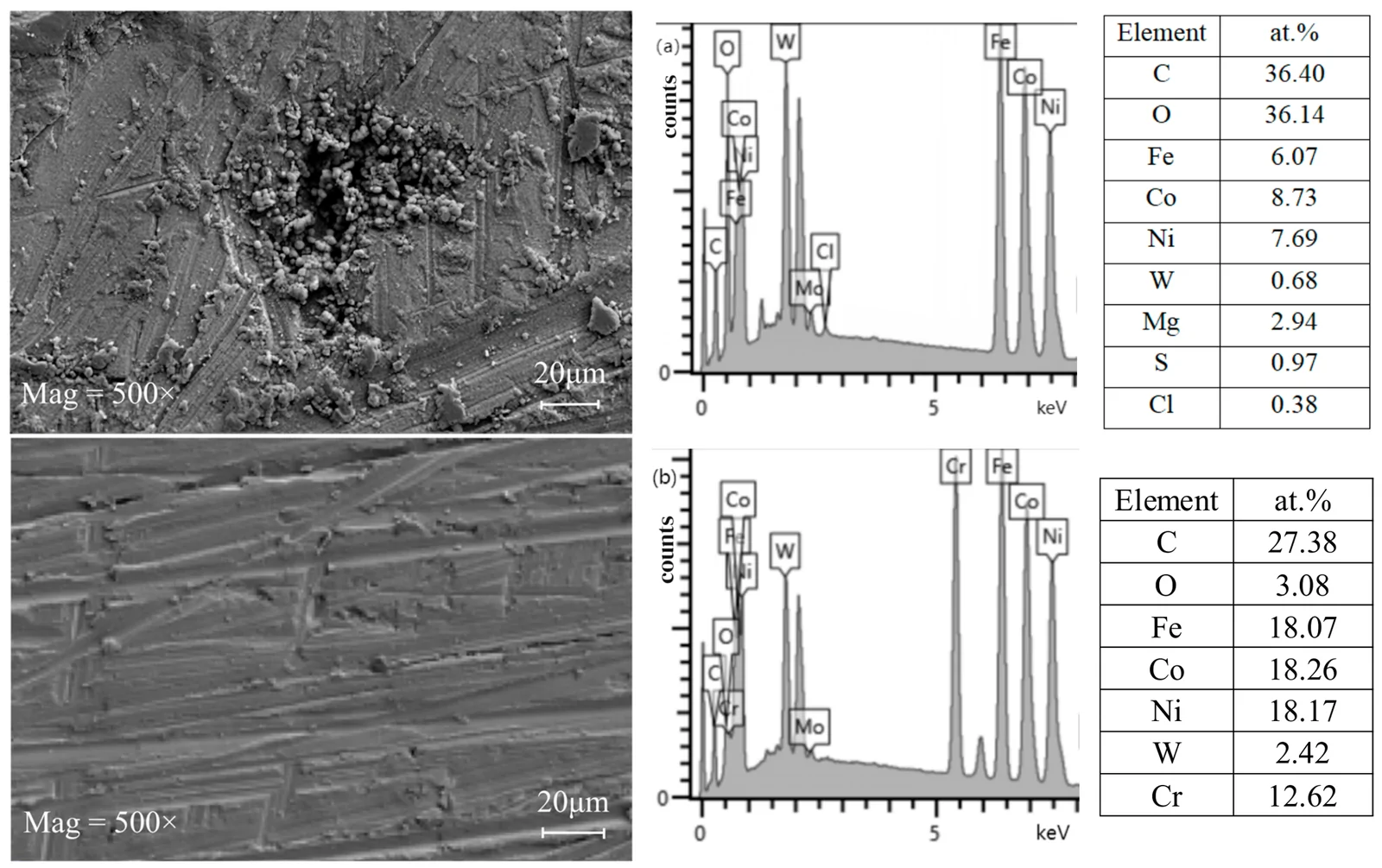
Surface micro-morphology and corresponding EDS analysis results of the CoFeNiMo_0.1_W_0.2_ and CoFeNiMo_0.1_W_0.2_Cr_0.7_ high-entropy alloys after 28 days of seawater immersion: (**a**) CoFeNiMo_0.1_W_0.2_ alloy (showing the pitted morphology); (**b**) CoFeNiMo_0.1_W_0.2_Cr_0.7_ alloy (showing the smooth ground surface).

**Figure 5:**
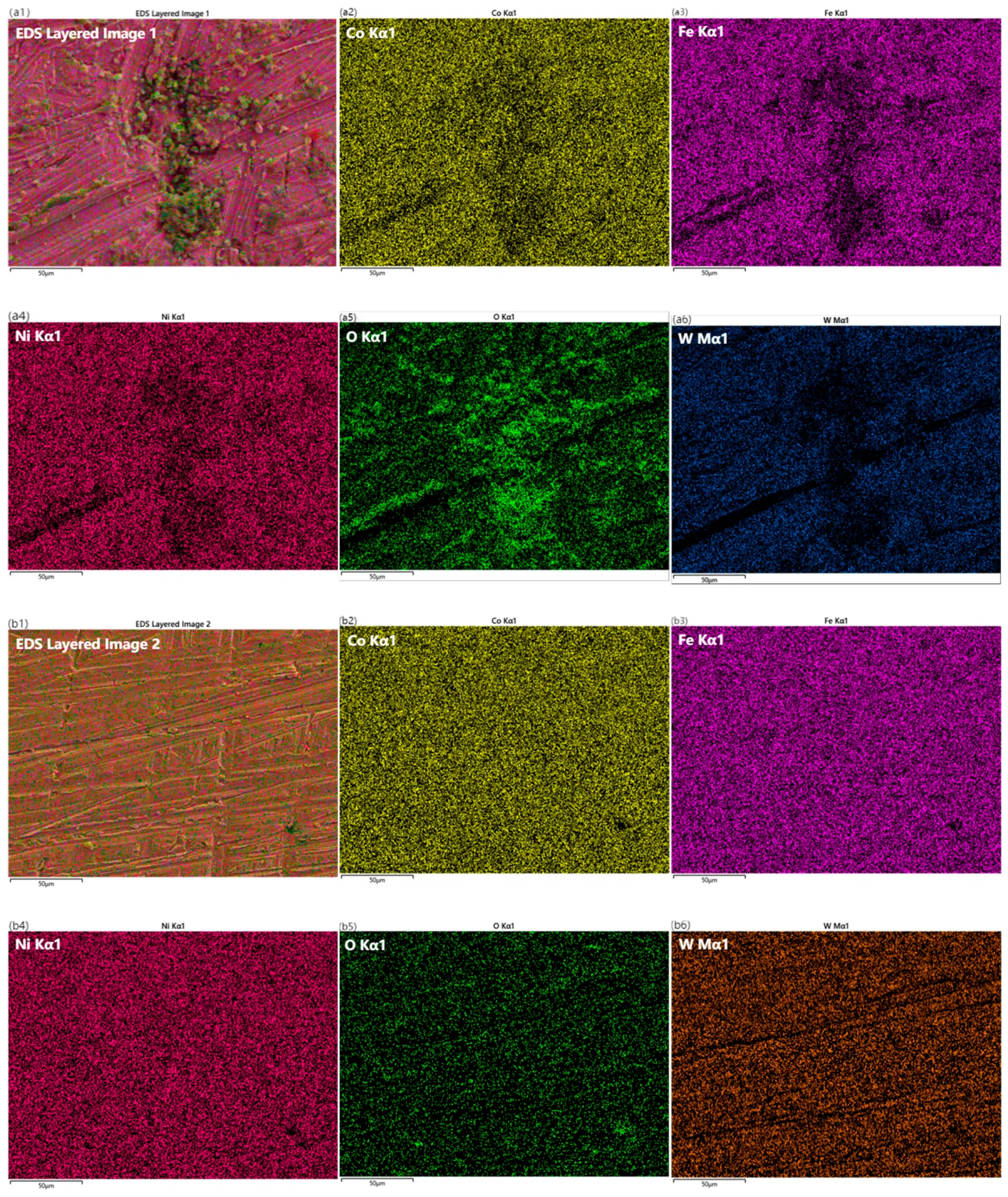
Surface EDS elemental distribution maps of two high-entropy alloys after 28 days of seawater immersion (EDS mapping). (**a**) CoFeNiMo_0.1_W_0.2_ alloy: (**a1**) layered elemental image; (**a2**–**a6**) show the elemental distribution maps of Co, Fe, Ni, O, and W, respectively. (**b**) CoFeNiMo_0.1_W_0.2_Cr_0.7_ alloy: (**b1**) layered elemental image; (**b2**–**b6**) show the elemental distribution maps of Co, Fe, Ni, O, and W, respectively.

After prolonged immersion in seawater, the surfaces of the two high-entropy alloys exhibited distinct corrosion product coverage on the CoFeNiMo_0.1_W_0.2_ sample, whereas the CoFeNiMo_0.1_W_0.2_Cr_0.7_ sample showed virtually no corrosion product coverage. The surface microstructures and EDS spectra are presented in Figure 4. On the CoFeNiMo_0.1_W_0.2_ surface, multiple pitting pits with diameters ranging from 50 to 80 μm were observed, each covered by granular corrosion products. EDS point scanning analysis revealed that the primary elements within these pits were Co, Fe, Ni, W, along with O, Mg, S, and Cl present in the seawater corrosion environment; notably, the oxygen content reached as high as 36.14 at.%, indicating the presence of substantial oxide products. In contrast, careful examination of the CoFeNiMo_0.1_W_0.2_Cr_0.7_ surface revealed no obvious pitting pits or signs of corrosion products; EDS results showed that its primary constituent elements were Co, Fe, Ni, W, and Cr, while the oxygen content was merely 3 at.%. The EDS element distribution maps, shown in Figure 5, further confirm the presence of oxide corrosion products on the CoFeNiMo_0.1_W_0.2_ surface, whereas the CoFeNiMo_0.1_W_0.2_Cr_0.7_ surface exhibited negligible oxide formation.

Consequently, it was concluded that the surface passive film of CoFeNiMo_0.1_W_0.2_ could not resist the attack of corrosive ions in seawater, leading to localized pitting corrosion. The accumulation of chloride ions within the pitting pits triggered autocatalytic hydrolysis and acidification, forming a typical galvanic cell [17,18], which exacerbated the severity of localized corrosion. As a result, a large number of pitting pits and localized deposits of corrosion products were observed on the alloy surface. In contrast, in the CoFeNiMo_0.1_W_0.2_Cr_0.7_ alloy, the chromium content reaches as high as 12.35 at.%, and the chromium-containing passive film exhibits excellent protective properties [19]. Therefore, no significant corrosion or oxidation products were observed on the alloy surface, and the metal remained in a passive state.

**Figure 6:**
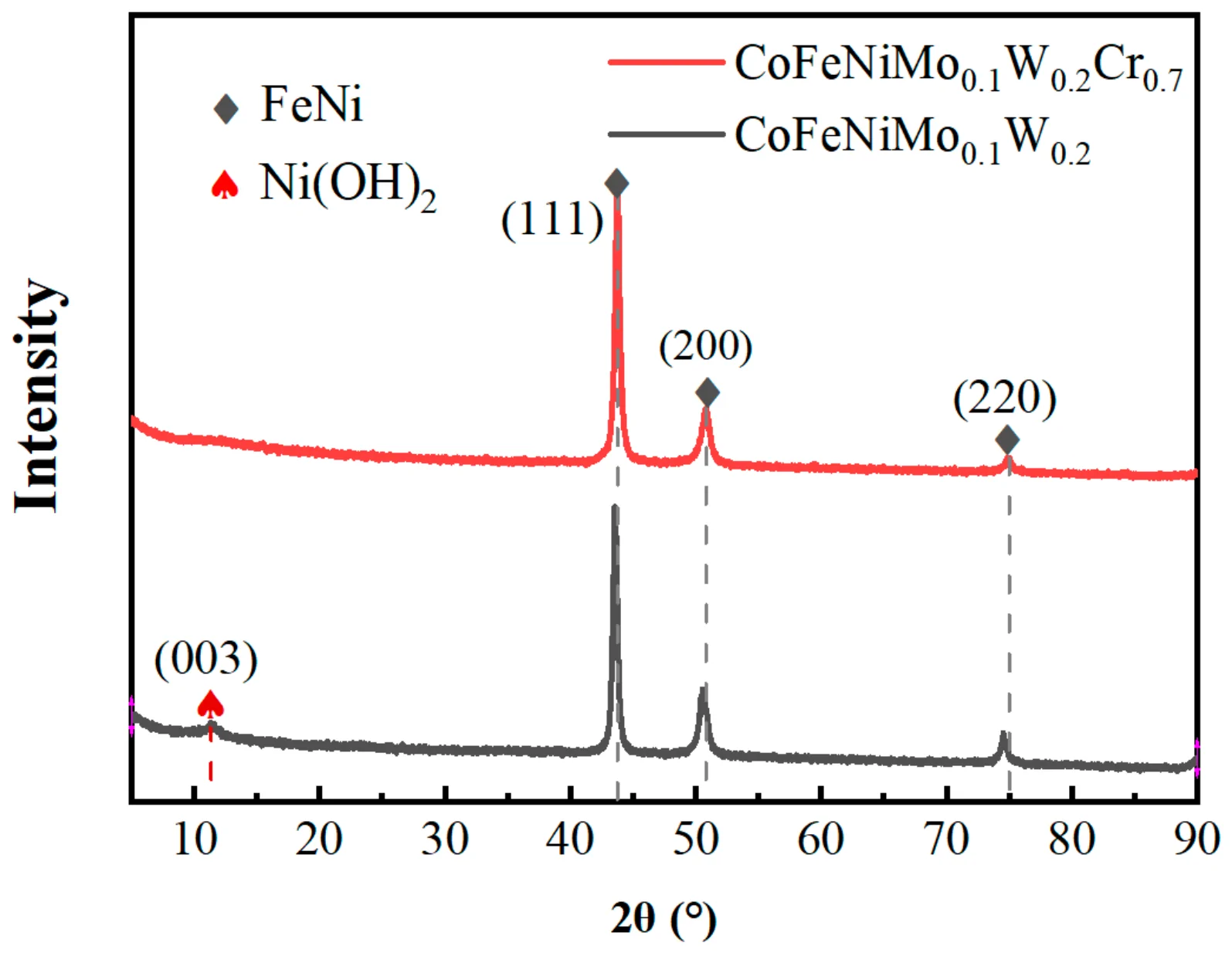
Surface XRD diffraction patterns of CoFeNiMo_0.1_W_0.2_ and CoFeNiMo_0.1_W_0.2_Cr_0.7_ high-entropy alloys after 28 days of immersion in seawater.

The surface phase compositions of two high-entropy alloys after 28 days of full immersion in seawater were analyzed using XRD, and the test results are shown in Figure 6. Both alloys exhibited distinct and sharp characteristic diffraction peaks near 2θ ≈ 44°, 51°, and 75°, corresponding to the (111), (200), and (220) diffraction planes of the face-centered cubic (FCC) FeNi solid solution phase (PDF#47-1405), respectively. This indicates that, whether in the Cr-free or high-Cr systems, the CoFeNiMo_0.1_W_0.2_ matrix consistently displays a typical single-phase FCC structure, with no significant Mo- or Cr-enriched precipitate phases observed. Furthermore, careful comparison of the diffraction peak positions reveals that the diffraction peaks of the Cr-containing alloys exhibit a slight shift toward higher angles compared to the Cr-free specimens. According to Bragg’s law 2*d*sin*θ* = *λ*, this shift is primarily attributed to the introduction of Cr atoms—which possess relatively smaller atomic radii—leading to a reduction in the interplanar spacing [20].

The key difference between the two high-entropy alloys lies in their behavior under prolonged immersion in seawater: the CoFeNiMo_0.1_W_0.2_ alloy exhibits a distinct diffraction peak at 2θ ≈ 11.4°, corresponding to the (003) characteristic peak of Ni(OH)_2_ (PDF#38-0715); additionally, its surface may contain amorphous oxides that are difficult to detect by XRD. In contrast, the CoFeNiMo_0.1_W_0.2_Cr_0.7_ alloy shows no additional diffraction peaks beyond those of its substrate. Consequently, the CoFeNiMo_0.1_W_0.2_ alloy undergoes active corrosion in seawater, yielding substantial corrosion products—a finding consistent with the EDS results.

### 3.3. Characterization of the Surface Passive Film

**Figure 7:**
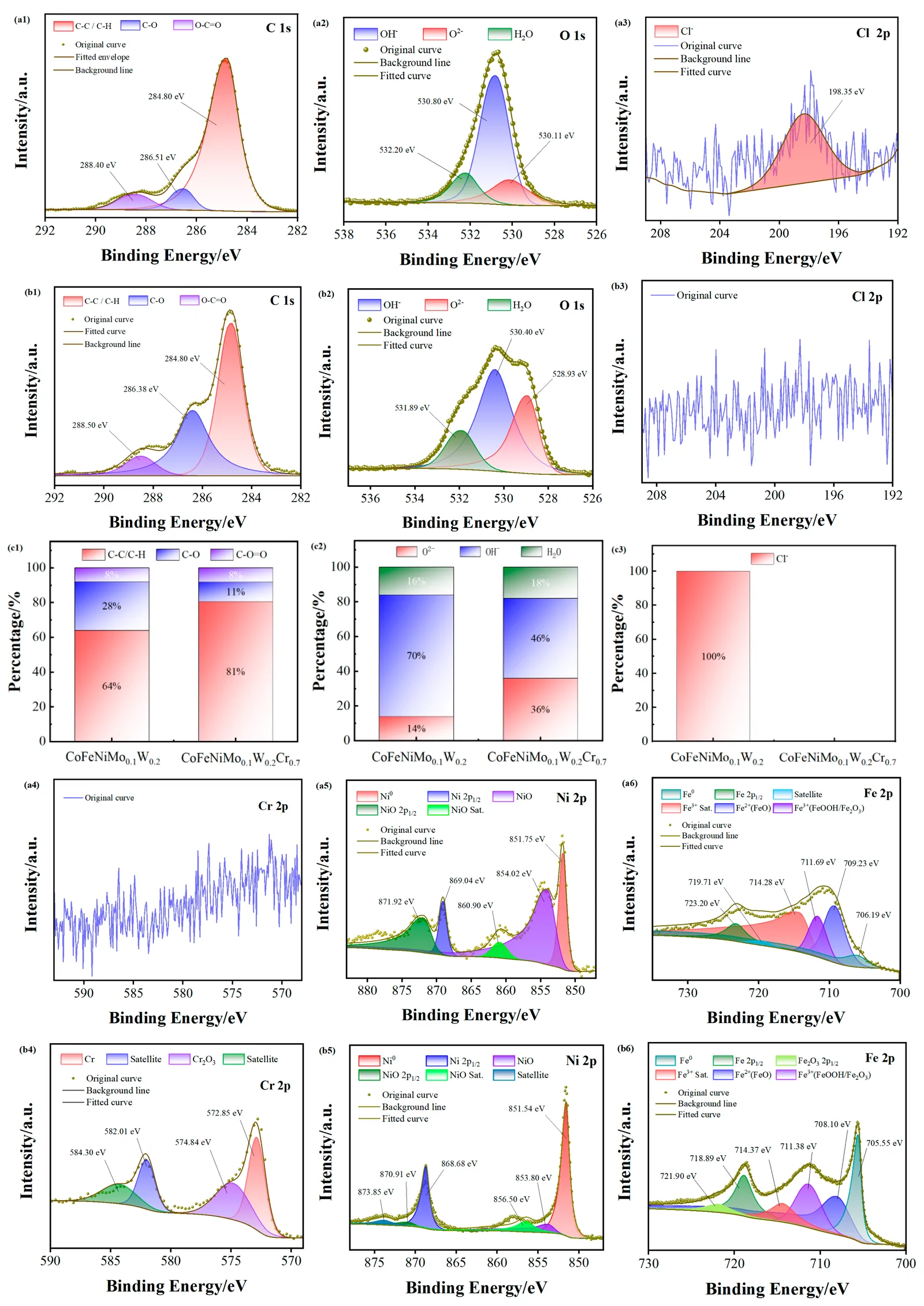

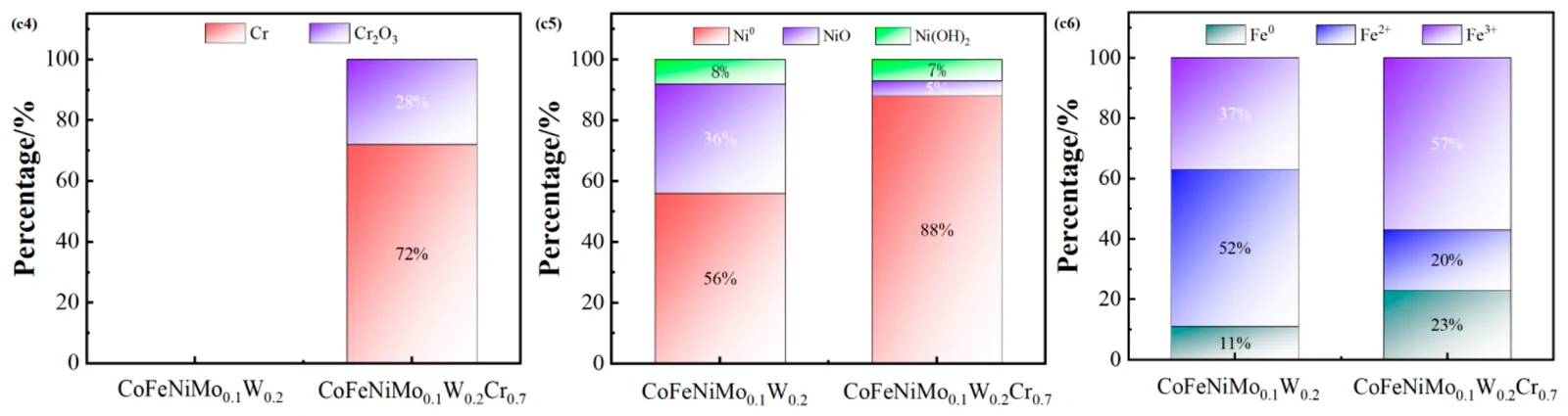
High-resolution XPS spectra and quantitative chemical state distributions of the passive films formed on CoFeNiMo_0.1_W_0.2_ and CoFeNiMo_0.1_W_0.2_Cr_0.7_ after 1 d of seawater immersion: (**a1**–**a6**) Deconvoluted XPS spectra of C 1s, O 1s, Cl 2p, Cr 2p, Ni 2p, and Fe 2p for CoFeNiMo_0.1_W_0.2_; (**b1**–**b6**) Deconvoluted XPS spectra of C 1s, O 1s, Cl 2p, Cr 2p, Ni 2p, and Fe 2p for CoFeNiMo_0.1_W_0.2_Cr_0.7_; (**c1**–**c6**) Corresponding relative fractions (calculated based on peak heights/areas) of the deconvoluted chemical species in C 1s, O 1s, Cl 2p, Cr 2p, Ni 2p, and Fe 2p, respectively.

To elucidate the chemical composition of the passive film on high-entropy alloys after immersion in seawater, as well as the mechanism by which the micro-alloying element Cr regulates the density of this passive film, high-resolution X-ray photoelectron spectroscopy (XPS) was employed to perform detailed narrow-band fitting and quantitative analysis on the surfaces of CoFeNiMo_0.1_W_0.2_ and CoFeNiMo_0.1_W_0.2_Cr_0.7_ specimens after 1 day of immersion in seawater; the results are presented in Figure 7.

All spectra were charge-corrected using the C-C/C-H binding energy (284.80 eV) of the contaminant carbon C 1s peak (Figure 7a1,b1); since the C signal is highly likely to originate from external contamination, no further detailed analysis was performed.

The high-resolution XPS spectra of O indicate that the surface film of CoFeNiMo_0.1_W_0.2_ consists predominantly of hydroxides, whereas that of CoFeNiMo_0.1_W_0.2_Cr_0.7_ consists predominantly of oxides; it is generally accepted that metal oxides are denser and offer superior protective properties compared to their hydrated hydroxide counterparts [21]. A distinct Cl signal was detected on the surface of CoFeNiMo_0.1_W_0.2_, indicating that in seawater environments, its surface passive film accumulates and adsorbs a large number of corrosive Cl^−^ ions; in contrast, no significant Cl signal was detected on the surface of the Cr-containing alloy CoFeNiMo_0.1_W_0.2_Cr_0.7_, which directly demonstrates that the introduction of Cr_2_O_3_ significantly enhances the barrier properties of the surface passive film, effectively inhibiting the penetration and adsorption of Cl^−^ ions from seawater into the passive film and the substrate interface [22]. Furthermore, a distinct Cr signal was detected on the surface of the Cr-containing alloy CoFeNiMo_0.1_W_0.2_Cr_0.7_, and the primary component of the passive film was identified as Cr_2_O_3_ [23]. Cr_2_O_3_ exhibits excellent electrical insulation properties and thermodynamic stability, suggesting its potential to further improve the protective performance of the passive film.

Distinct Ni and Fe signals were also detected in the surface films of both alloys; in CoFeNiMo_0.1_W_0.2_, Ni oxide was the dominant phase, whereas in CoFeNiMo_0.1_W_0.2_Cr_0.7_, the contents of Ni oxide and Ni hydroxide showed little difference; in CoFeNiMo_0.1_W_0.2_, the Fe-phase oxide was predominantly composed of lower-valence Fe^2+^, whereas in CoFeNiMo_0.1_W_0.2_Cr_0.7_, the Fe-phase in the oxide film was predominantly composed of Fe^3+^ [24].

Overall, upon the introduction of Cr into CoFeNiMo_0.1_W_0.2_, a substantial amount of Cr_2_O_3_ oxide was formed within the passive film, which inhibited the penetration of chloride ions into the interior of the passive film, thereby endowing the alloy with excellent spontaneous passivation corrosion resistance properties.

## 4. Conclusions

This study investigated the long-term seawater corrosion behavior and passivation mechanisms of CoFeNiMo_0.1_W_0.2_ and CoFeNiMo_0.1_W_0.2_Cr_0.7_ high-entropy alloys. The main conclusions are as follows:

(1) The addition of Cr significantly improves the thermodynamic stability and electrochemical impedance of the alloy in seawater. During the 49-day immersion, the OCP of the Cr-containing alloy shifted positively by 300–400 mV, and its charge transfer resistance (R_ct_) increased by nearly two orders of magnitude compared to the base alloy, exhibiting stable capacitive behavior.
(2) The Cr-free CoFeNiMo_0.1_W_0.2_ alloy suffers from severe localized pitting and active dissolution, accompanied by the accumulation of crystalline Cr_2_O_3_ corrosion products. In contrast, the CoFeNiMo_0.1_W_0.2_Cr_0.7_ alloy maintains a smooth, pit-free surface with a stable single-phase FCC structure.
(3) XPS analysis reveals that the incorporation of Cr promotes the formation of a dense, Cr_2_O_3_-enriched passive film that contains a higher proportion of protective oxides and lower hydroxides. This film effectively hinders the adsorption and penetration of aggressive Cl^−^ ions, endowing the alloy with superior long-term marine corrosion resistance [25].

## Figures and Tables

**Table 1:** EIS fitting parameters of CoFeNiMo_0.1_W_0.2_ high-entropy alloy in seawater with different immersion times.

Metal	Time	R_s_	Q_f_		R_f_	Q_dl_		R_ct_	W
	(d)	(Ω·cm^2^)	Y_01_ (s^n1^·cm^−2^)	n_1_	(Ω·cm^2^)	Y_02_ (s^n2^·cm^−2^)	n_2_	(Ω·cm^2^)	(S·s^0.5^)
CoFeNiMo_0.1_W_0.2_	1	8.27 ± 0.45	(8.42 ± 0.52) × 10^−5^	0.76 ± 0.02	6.35 ± 0.38	(1.26 ± 0.15) × 10^−4^	0.73 ± 0.05	(4.13 ± 0.25) × 10^3^	(1.95 ± 0.12) × 10^−3^
	2	8.04 ± 0.42	(7.48 ± 0.45) × 10^−5^	0.77 ± 0.04	7.68 ± 0.46	(1.32 ± 0.35) × 10^−4^	0.73 ± 0.08	(5.49 ± 0.31) × 10^3^	(2.11 ± 0.14) × 10^−3^
	3	7.34 ± 0.39	(9.72 ± 0.58) × 10^−5^	0.74 ± 0.01	20.39 ± 1.25	(1.20 ± 0.82) × 10^−4^	0.72 ± 0.15	(5.10 ± 0.28) × 10^3^	(1.58 ± 0.09) × 10^−3^
	7	8.02 ± 0.45	(7.67 ± 0.48) × 10^−5^	0.72 ± 0.05	28.71 ± 1.62	(1.10 ± 0.18) × 10^−4^	0.74 ± 0.03	(8.20 ± 0.45) × 10^3^	(1.53 ± 0.08) × 10^–3^
	14	9.76 ± 0.52	(5.69 ± 0.34) × 10^−5^	0.74 ± 0.12	29.78 ± 1.70	(1.17 ± 1.45) × 10^−4^	0.74 ± 0.18	(1.10 ± 0.06) × 10^4^	(1.27 ± 0.07) × 10^−3^
	21	6.24 ± 0.33	(8.14 ± 0.49) × 10^−5^	0.7 ± 0.03	48.97 ± 2.80	(1.07 ± 0.22) × 10^−4^	0.76 ± 0.06	(6.95 ± 0.38) × 10^3^	(6.30 ± 0.35) × 10^−4^
	28	5.94 ± 0.31	(5.55 ± 0.32) × 10^−5^	0.71 ± 0.03	29.45 ± 1.65	(1.40 ± 1.90) × 10^−4^	0.79 ± 0.12	(7.30 ± 0.40) × 10^3^	(1.82 ± 0.10) × 10^−3^
	49	7.32 ± 0.38	(6.84 ± 0.41) × 10^−5^	0.64 ± 0.18	74.18 ± 4.10	(1.20 ± 0.07) × 10^−4^	0.83 ± 0.04	(1.48 ± 0.08) × 10^4^	(7.48 ± 0.40) × 10^−4^
CoFeNiMo_0.1_W_0.2_Cr_0.7_	1	7.98 ± 0.42	(2.02 ± 0.12) × 10^−5^	0.94 ± 0.05	5.95 ± 0.30	(1.61 ± 0.09) × 10^−5^	0.91 ± 0.05	(5.25 ± 0.28) × 10^6^	2.17 ± ×10^−6^
	2	7.04 ± 0.37	(1.49 ± 0.09) × 10^−5^	0.99 ± 0.03	6.27 ± 0.32	(3.60 ± 0.20) × 10^−5^	0.86 ± 0.08	(1.17 ± 0.06) × 10^6^	-
	3	7.06 ± 0.37	(2.66 ± 0.15) × 10^−5^	0.93 ± 0.12	10.88 ± 0.55	(1.66 ± 0.09) × 10^−5^	0.90 ± 0.04	(2.48 ± 0.13) × 10^6^	(3.03 ± 0.16) × 10^−6^
	7	7.06 ± 0.37	(2.66 ± 0.15) × 10^−5^	0.93 ± 0.04	10.88 ± 0.55	(1.66 ± 0.09) × 10^−5^	0.90 ± 0.15	(2.48 ± 0.13) × 10^6^	(3.03 ± 0.16) × 10^−6^
	14	7.09 ± 0.38	(2.28 ± 0.13) × 10^−5^	0.93 ± 0.08	13.51 ± 0.68	(1.21 ± 0.06) × 10^−5^	0.90 ± 0.06	(2.40 ± 0.12) × 10^6^	(1.70 ± 0.09) × 10^−6^
	21	7.45 ± 0.40	(1.52 ± 0.08) × 10^−5^	0.96 ± 0.02	7.71 ± 0.39	(2.21 ± 0.12) × 10^−5^	0.87 ± 0.05	(1.72 ± 0.09) × 10^6^	(8.21 ± 0.45) × 10^−16^
	28	5.86 ± 0.30	(2.40 ± 0.13) × 10^−5^	0.93 ± 0.15	38.71 ± 1.90	(8.91 ± 0.45) × 10^−6^	0.90 ± 0.11	(4.19 ± 0.22) × 10^6^	(1.44 ± 0.08) × 10^−6^
	49	7.58 ± 0.40	(1.93 ± 0.10) × 10^−5^	0.93 ± 0.06	13.65 ± 0.70	(8.29 ± 0.42) × 10^−6^	0.91 ± 0.08	(1.27 ± 0.07) × 10^6^	(2.21 ± 0.12) × 10^−6^

**Table 2:** Equivalent corrosion current density (*i*_corr_) of CoFeNiMo_0.1_W_0.2_ and CoFeNiMo_0.1_W_0.2_Cr_0.7_ high-entropy alloys in seawater at different immersion times (estimated from *R*_ct_ via the Stern-Geary equation).

Immersion Time	CoFeNiMo_0.1_W_0.2_		CoFeNiMo_0.1_W_0.2_Cr_0.7_	
(d)	R_ct_ (Ω·cm^2^)	i_corr_ (μA·cm^−2^)	R_ct_ (Ω·cm^2^)	i_corr_ (μA·cm^−2^)
1	4.13 × 10^3^	6.30	5.25 × 10^6^	0.00495
2	5.49 × 10^3^	4.74	1.17 × 10^6^	0.0222
3	5.10 × 10^3^	5.10	2.48 × 10^6^	0.0105
7	8.20 × 10^3^	3.17	2.48 × 10^6^	0.0105
14	1.10 × 10^4^	2.36	2.40 × 10^6^	0.0108
21	6.95 × 10^3^	3.74	1.72 × 10^6^	0.0151
28	7.30 × 10^3^	3.56	4.19 × 10^6^	0.0062
49	1.48 × 10^4^	1.76	1.27 × 10^6^	0.0205

## Data Availability

The original contributions presented in this study are included in the article. Further inquiries can be directed to the corresponding author.
